# Efficacy and safety of high-flow oxygen therapy application for chronic obstructive pulmonary disease with acute hypercapnic respiratory failure: A protocol for systematic review and meta-analysis

**DOI:** 10.1097/MD.0000000000025489

**Published:** 2021-04-16

**Authors:** Mingxu Zheng, Liangliang Dong, Zerui Hao, Shuyun Wang

**Affiliations:** Department of respiratory, Liaocheng Second People's Hospital, Liaocheng, Shandong, China.

**Keywords:** acute hypercapnic respiratory failure, chronic obstructive pulmonary disease, high-flow oxygen therapy, meta-analysis, noninvasive ventilation, protocol

## Abstract

**Background:**

Presently, there are no reviews or meta-analyses comparing the efficacy and safety of high-flow oxygen therapy (HFOT) and noninvasive ventilation (NIV) as first-line treatment in exacerbated chronic obstructive pulmonary disease (COPD) patients. The present protocol is conceived to evaluate whether HFOT is noninferior to NIV in treatment of patients with COPD and acute hypercapnic respiratory failure.

**Methods:**

We will follow the Preferred Reporting Items for Systematic Reviews and Meta-Analyses reporting guidelines and the recommendations of the Cochrane Collaboration to conduct this meta-analysis. Reviewers will search the PubMed, Cochrane Library, Web of Science, and EMBASE online databases using the key phrases “high-flow oxygen therapy,” “chronic obstructive pulmonary disease,” and “acute hypercapnic respiratory failure” for all English-language cohort studies published up to April, 2021. The cohort studies focusing on assess the efficacy and safety of HFOT and NIV in the treatment of patients with COPD and acute hypercapnic respiratory failure will be included in our meta-analysis. The primary outcome is treatment failure, whereas the secondary outcomes included arterial blood gas analysis, dyspnea score, comfort score, mortality, and total ICU and hospital lengths of stay.

**Results:**

The trial is conducted to test the hypothesis that HFOT, administered immediately after extubation, is not inferior to the NIV in reducing the rate of treatment failure in patients with COPD who were previously intubated due to hypercapniac respiratory failure.

**Registration number:**

10.17605/OSF.IO/Z2PEJ.

## 1 Introduction

Chronic obstructive pulmonary disease (COPD) is the fourth leading cause of death worldwide.^[1]^ By 2030, COPD will be number three according to the World Health Organization.^[2]^ Restricted airflow and insufficient alveolar ventilation impair arterial oxygen exchange in patients with COPD.^[3]^ As the disease progresses, hypoxemia and hypercapnia, caused by obstruction of the peripheral airways, lead to reduced pulmonary gas exchange capacity, disruption of lung parenchyma, and pulmonary vascular abnormalities, increasing the frequency of acute exacerbations. Patients with chronic respiratory failure and hypercapnia suffer from severe breathing difficulties, reduced quality of life, and even increased mortality.^[4]^

Multiple studies have shown that a sequential strategy of noninvasive ventilation (NIV) using the pulmonary infection control window as an entry point can reduce the duration of invasive ventilation and significantly improve outcomes in patients with COPD.^[5,6]^ However, due to poor patient tolerance, NIV is ineffective in approximately 15% to 25% of patients and may result in endotracheal intubation. Additional respiratory support is urgently needed for extubated COPD patients who are intolerant to NIV or contraindications to NIV.^[7,8]^

High-flow oxygen therapy (HFOT) is a more recent treatment in which a mixture of heated and humidified air oxygen is delivered at a high flow rate through a large-bore nasal catheter.^[9]^ Compared with standard oxygen therapy, HFOT can cleanse the anatomical dead space of the upper respiratory tract, generate a certain amount of exhalopharyngeal pressure proportional to flow, thereby increasing end-expiratory lung volume, reducing breathing rate by reducing breathing time, facilitating clearance of tracheobronchial secretions, and reducing inspiratory effort.^[10,11]^

Presently, there are no reviews or meta-analyses comparing the efficacy and safety of HFOT and NIV as first-line treatment in exacerbated COPD patients. The present protocol is conceived to evaluate whether HFOT is noninferior to NIV in treatment of patients with COPD and acute hypercapnic respiratory failure. The trial is conducted to test the hypothesis that HFOT, administered immediately after extubation, is no inferior to the NIV in reducing the rate of treatment failure in patients with COPD who were previously intubated due to hypercapniac respiratory failure.

## 2 Materials and methods

### 2.1 Search strategy

**Figure 1:**
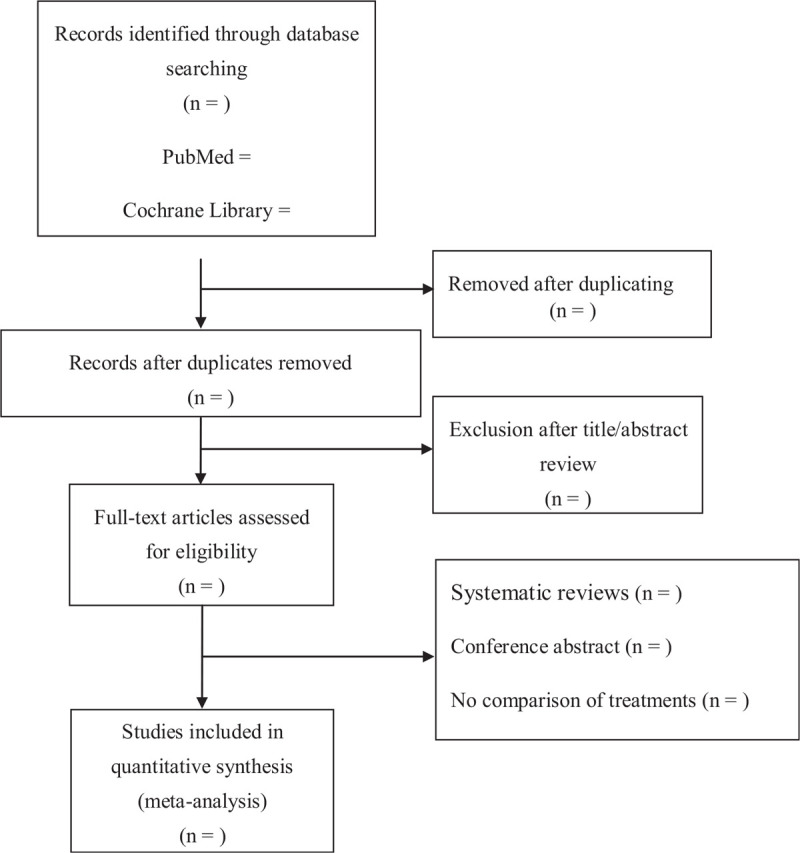
Flow diagram of study identification.

We will follow the Preferred Reporting Items for Systematic Reviews and Meta-Analyses (PRISMA) reporting guidelines and the recommendations of the Cochrane Collaboration to conduct this meta-analysis. The systematic review protocol has been registered on Open Science Framework registries (https://osf.io/z2pej). The registration number is 10.17605/OSF.IO/Z2PEJ. The detailed guidelines can be found at www.prisma-statement.org. Reviewers will search the PubMed, Cochrane Library, Web of Science, and EMBASE online databases using the key phrases “high-flow oxygen therapy,” “chronic obstructive pulmonary disease,” and “acute hypercapnic respiratory failure” for all English-language cohort studies published up to April, 2021. Ethical approval is not necessary because the present meta-analysis will be performed based on previous published studies. Flow diagram of study identification is shown in Figure 1.

### 2.2 Eligibility criteria

The cohort studies focusing on assess the efficacy and safety of HFOT and NIV in the treatment of patients with COPD and acute hypercapnic respiratory failure will be included in our meta-analysis. At least one of the following outcomes should have been measured: treatment failure (defined as a return to invasive mechanical ventilation, or a switch in respiratory support modality), arterial blood gas analysis, dyspnea score, comfort score, mortality, and total ICU and hospital lengths of stay. The exclusion criteria contain biochemical trials, reviews, case reports, no assessment of outcomes mentioned above, and no comparison of HFOT and NIV.

### 2.3 Data extraction

Data will be extracted from the included studies by 2 independent reviewers. Relevant data extracted from the original studies will include author, publication year, study design; patient demographic details such as patients’ number, average age, body mass index, and sex ratio. The primary outcome is treatment failure, whereas the secondary outcomes included arterial blood gas analysis, dyspnea score, comfort score, mortality, and total ICU and hospital lengths of stay. If the data cannot be extracted directly, we will contact the authors for more information. Otherwise, we will extract them from figures or calculate them with the guideline of Cochrane Handbook for Systematic Reviews of Interventions 5.1.0.

### 2.4 Data analysis

Review Manager software (v 5.3; Cochrane Collaboration) is used for the meta-analysis. Extracted data are entered into Review Manager by the first independent author and checked by the second independent author. Risk ratio with a 95% confidence interval (CI) or standardized mean difference with 95% CI are assessed for dichotomous outcomes or continuous outcomes, respectively. The heterogeneity is assessed by using the *Q* test and *I*^2^ statistic. An *I*^2^ value of <25% is chosen to represent low heterogeneity and an *I*^2^ value of >75% to indicate high heterogeneity. All outcomes are pooled on random-effect model. A *P* value of <.05 is considered to be statistically significant.

### 2.5 Assessment of methodological quality

To achieve a consistency (at least 80%) of risk of bias assessment, the risk of bias assessors will pre-assess a sample of eligible studies. Results of the pilot risk of bias will be discussed among review authors and assessors. Two independent reviewers will assess the risk of bias of the included studies at study level. We will follow the guidance in the latest version of Cochrane Handbook for systematic reviews of interventions when choosing and using tools to assessing risk of bias for randomized trials (version 2 of the Cochrane risk of bias tool for randomized trials, RoB 2) and nonrandomized trials (the Risk Of Bias In Non-randomized Studies of Interventions, ROBINS-I tool). Any disagreements will be discussed and resolved in discussion with a third reviewer. Studies with high risk of bias or unclear bias will be given less weight in our data synthesis.

## 3 Discussion

HFOT consists of a totally conditioned, warmed, and humidified air/oxygen blend through a wide-bore nasal cannula at a flow rate between 20 and 60 L/min. Compared with the “conventional" oxygen therapy devices, which deliver gas at 5 to 20 L/min, during HFNC the tracheal inspiratory oxygen fraction is more predictable and the mucociliary function is better preserved.^[7]^ presently, there are no reviews or meta-analyses comparing the efficacy and safety of HFOT and NIV as first-line treatment in exacerbated COPD patients. The present protocol is conceived to evaluate whether HFOT is noninferior to NIV in treatment of patients with COPD and acute hypercapnic respiratory failure. The trial is conducted to test the hypothesis that HFOT, administered immediately after extubation, is no inferior to the NIV in reducing the rate of treatment failure in patients with COPD who were previously intubated due to hypercapniac respiratory failure.

## Author contributions

**Conceptualization:** Zerui Hao.

**Data curation:** Mingxu Zheng, Liangliang Dong.

**Formal analysis:** Mingxu Zheng, Liangliang Dong.

**Funding acquisition:** Shuyun Wang.

**Investigation:** Mingxu Zheng, Liangliang Dong.

**Methodology:** Liangliang Dong, Zerui Hao.

**Project administration:** Shuyun Wang.

**Resources:** Shuyun Wang.

**Software:** Liangliang Dong, Zerui Hao.

**Supervision:** Shuyun Wang.

**Validation:** Mingxu Zheng, Liangliang Dong, Zerui Hao.

**Visualization:** Zerui Hao.

**Writing – original draft:** Mingxu Zheng.

**Writing – review & editing:** Zerui Hao, Shuyun Wang.
